# Dr. Henry James Stokes

**Published:** 1911-04-22

**Authors:** 


### OBITUARY.

After long illness Dr. Henry James Stokes has died at Highbury, in his eighty-ninth year. His medical education was obtained at University College, London, and at Edinburgh University. He took his M.R.C.S. in 1843, his L.S.A. in 1844, and graduated M.D. of Edinburgh in 1845.

